# Tetra­methyl 4,4′-carbonyl­bis(benzene-1,2-dicarboxyl­ate)

**DOI:** 10.1107/S1600536809009271

**Published:** 2009-03-19

**Authors:** Xin-Yi Zhu, Guo-Wei Gao, Jian Men, Seik Weng Ng

**Affiliations:** a College of Chemistry, Sichuan University, Chengdu 610064, People’s Republic of China; b Department of Chemistry, University of Malaya, 50603 Kuala Lumpur, Malaysia

## Abstract

In the mol­ecule of the title compound, C_21_H_18_O_9_, the two aromatic rings are aligned at an angle of 49.7 (1)°.

## Related literature

For the parent acid monohydrate, see: Fitzgerald & Gerkin (1997 ▶). For related literature, see: Zhang *et al.* (2004 ▶).
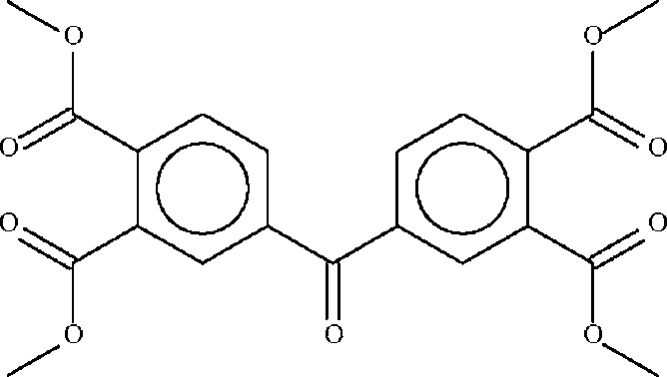


## Experimental

### 

#### Crystal data

C_21_H_18_O_9_
*M* *_r_* = 414.35
Monoclinic, 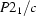

*a* = 11.8627 (2) Å
*b* = 17.3678 (3) Å
*c* = 9.2506 (2) Å
β = 95.150 (1)°
*V* = 1898.20 (6) Å^3^
*Z* = 4
Mo *K*α radiation
μ = 0.12 mm^−1^
*T* = 123 K
0.45 × 0.15 × 0.05 mm

#### Data collection

Bruker SMART APEX diffractometer
Absorption correction: none
14279 measured reflections
4339 independent reflections
3596 reflections with *I* > 2σ(*I*)
*R*_int_ = 0.024

#### Refinement

*R*[*F* ^2^ > 2σ(*F* ^2^)] = 0.050
*wR*(*F* ^2^) = 0.139
*S* = 1.04
4339 reflections
275 parameters
H-atom parameters constrained
Δρ_max_ = 0.89 e Å^−3^
Δρ_min_ = −0.50 e Å^−3^

### 

Data collection: *APEX2* (Bruker, 2008 ▶); cell refinement: *SAINT* (Bruker, 2008 ▶); data reduction: *SAINT*; program(s) used to solve structure: *SHELXS97* (Sheldrick, 2008 ▶); program(s) used to refine structure: *SHELXL97* (Sheldrick, 2008 ▶); molecular graphics: *X-SEED* (Barbour, 2001 ▶); software used to prepare material for publication: *publCIF* (Westrip, 2009 ▶).

## Supplementary Material

Crystal structure: contains datablocks global, I. DOI: 10.1107/S1600536809009271/tk2392sup1.cif

Structure factors: contains datablocks I. DOI: 10.1107/S1600536809009271/tk2392Isup2.hkl

Additional supplementary materials:  crystallographic information; 3D view; checkCIF report

## Figures and Tables

**Fig. 1.**
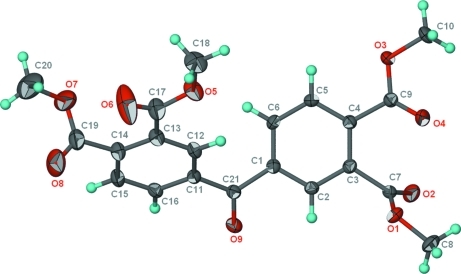
Thermal ellipsoid plot (Barbour, 2001) of C21H18O9 at the 70% probability level; hydrogen atoms are drawn as spheres of arbitrary radius.

## supplementary crystallographic information

### Experimental

3,3',4,4'-Tetracarboxybenzophenone (29.4 g, 0.1 mol) and *p*-toluenesulfonic acid (2.0 g, 0.01 mol) were heated in a mixture of toluene (100 ml) and methanol (50 ml) in a Dean-Stark apparatus for 20 h. Water (500 ml) was added to the mixture, the organic phase was separated and washed with saturated sodium carbonate. The toluene was removed and the product purified from ethanol to afford a white powder (34.7 g, 90% yield; m.p. 382–384 K). Colorless single crystals were obtained by recrystallization from toluene.

### Refinement

Carbon-bound H-atoms were placed in calculated positions (C—H 0.95 to 0.98 Å) and were included in the refinement in the riding model approximation, with *U*(H) set to 1.2 to 1.5*U*(C).

### Crystal data

**Table:**

C_21_H_18_O_9_	*F*(000) = 864
*M**_r_* = 414.35	*D*_x_ = 1.450 Mg m^−^^3^
Monoclinic, *P*2_1_/*c*	Mo *K*α radiation, λ = 0.71073 Å
Hall symbol: -P 2ybc	Cell parameters from 5155 reflections
*a* = 11.8627 (2) Å	θ = 2.9–28.2°
*b* = 17.3678 (3) Å	µ = 0.12 mm^−^^1^
*c* = 9.2506 (2) Å	*T* = 123 K
β = 95.150 (1)°	Block, colorless
*V* = 1898.20 (6) Å^3^	0.45 × 0.15 × 0.05 mm
*Z* = 4	

### Data collection

**Table:**

Bruker SMART APEX [APEXII?] diffractometer	3596 reflections with *I* > 2σ(*I*)
Radiation source: fine-focus sealed tube	*R*_int_ = 0.024
graphite	θ_max_ = 27.5°, θ_min_ = 1.7°
ω scans	*h* = −15→15
14279 measured reflections	*k* = −22→22
4339 independent reflections	*l* = −11→12

### Refinement

**Table:**

Refinement on *F*^2^	Primary atom site location: structure-invariant direct methods
Least-squares matrix: full	Secondary atom site location: difference Fourier map
*R*[*F*^2^ > 2σ(*F*^2^)] = 0.050	Hydrogen site location: inferred from neighbouring sites
*wR*(*F*^2^) = 0.139	H-atom parameters constrained
*S* = 1.04	*w* = 1/[σ^2^(*F*_o_^2^) + (0.0704*P*)^2^ + 1.4144*P*] where *P* = (*F*_o_^2^ + 2*F*_c_^2^)/3
4339 reflections	(Δ/σ)_max_ = 0.001
275 parameters	Δρ_max_ = 0.89 e Å^−^^3^
0 restraints	Δρ_min_ = −0.50 e Å^−^^3^

### Fractional atomic coordinates and isotropic or equivalent isotropic displacement parameters (Å^2^)

**Table:**

	*x*	*y*	*z*	*U*_iso_*/*U*_eq_	
O1	0.82828 (10)	0.56029 (7)	0.93784 (12)	0.0205 (3)	
O2	1.01037 (11)	0.56968 (8)	0.88890 (13)	0.0261 (3)	
O3	0.89532 (11)	0.62457 (7)	0.42114 (13)	0.0227 (3)	
O4	0.87735 (13)	0.65645 (7)	0.65258 (14)	0.0288 (3)	
O5	0.47658 (11)	0.33391 (8)	0.30590 (16)	0.0310 (3)	
O6	0.38426 (16)	0.22311 (10)	0.2777 (3)	0.0726 (7)	
O7	0.50941 (16)	0.09165 (11)	0.19849 (17)	0.0525 (5)	
O8	0.42373 (14)	0.05561 (10)	0.38926 (19)	0.0458 (4)	
O9	0.85745 (11)	0.25995 (7)	0.79479 (13)	0.0236 (3)	
C1	0.82935 (13)	0.36953 (9)	0.64550 (17)	0.0163 (3)	
C2	0.85873 (13)	0.42065 (9)	0.75874 (17)	0.0165 (3)	
H2	0.8650	0.4026	0.8561	0.020*	
C3	0.87895 (13)	0.49763 (9)	0.73051 (17)	0.0160 (3)	
C4	0.87127 (13)	0.52414 (9)	0.58682 (17)	0.0158 (3)	
C5	0.84754 (14)	0.47205 (9)	0.47349 (17)	0.0180 (3)	
H5	0.8463	0.4892	0.3759	0.022*	
C6	0.82574 (14)	0.39516 (9)	0.50221 (17)	0.0177 (3)	
H6	0.8084	0.3602	0.4245	0.021*	
C7	0.91496 (14)	0.54819 (9)	0.85827 (17)	0.0173 (3)	
C8	0.85333 (17)	0.60385 (11)	1.07015 (19)	0.0262 (4)	
H8A	0.7826	0.6222	1.1053	0.039*	
H8B	0.9012	0.6480	1.0508	0.039*	
H8C	0.8932	0.5709	1.1440	0.039*	
C9	0.88242 (14)	0.60863 (9)	0.55979 (17)	0.0170 (3)	
C10	0.90221 (18)	0.70612 (10)	0.3881 (2)	0.0280 (4)	
H10A	0.8282	0.7302	0.3952	0.042*	
H10B	0.9246	0.7126	0.2894	0.042*	
H10C	0.9585	0.7306	0.4573	0.042*	
C11	0.72742 (14)	0.23959 (9)	0.59007 (17)	0.0168 (3)	
C12	0.63715 (14)	0.27188 (9)	0.50347 (18)	0.0187 (3)	
H12	0.6268	0.3261	0.5024	0.022*	
C13	0.56220 (14)	0.22528 (10)	0.41868 (19)	0.0210 (4)	
C14	0.57621 (15)	0.14533 (10)	0.42228 (19)	0.0220 (4)	
C15	0.66549 (15)	0.11274 (10)	0.50977 (19)	0.0223 (4)	
H15	0.6748	0.0584	0.5127	0.027*	
C16	0.74083 (14)	0.15960 (9)	0.59265 (18)	0.0189 (3)	
H16	0.8019	0.1372	0.6515	0.023*	
C17	0.46489 (16)	0.25921 (11)	0.3262 (2)	0.0287 (4)	
C18	0.38474 (18)	0.37057 (13)	0.2166 (2)	0.0354 (5)	
H18A	0.3787	0.3477	0.1194	0.053*	
H18B	0.4001	0.4258	0.2094	0.053*	
H18C	0.3136	0.3629	0.2609	0.053*	
C19	0.49327 (17)	0.09303 (11)	0.3372 (2)	0.0300 (4)	
C20	0.4158 (3)	0.0543 (2)	0.1117 (3)	0.0687 (9)	
H20A	0.4090	0.0008	0.1436	0.103*	
H20B	0.4303	0.0552	0.0091	0.103*	
H20C	0.3452	0.0819	0.1242	0.103*	
C21	0.80855 (14)	0.28732 (9)	0.68492 (17)	0.0172 (3)	

### Atomic displacement parameters (Å^2^)

**Table:**

	*U*^11^	*U*^22^	*U*^33^	*U*^12^	*U*^13^	*U*^23^
O1	0.0222 (6)	0.0240 (6)	0.0151 (5)	0.0013 (5)	−0.0003 (4)	−0.0048 (5)
O2	0.0248 (7)	0.0309 (7)	0.0223 (6)	−0.0094 (5)	0.0005 (5)	−0.0043 (5)
O3	0.0370 (7)	0.0143 (6)	0.0168 (6)	−0.0020 (5)	0.0027 (5)	0.0017 (4)
O4	0.0485 (9)	0.0173 (6)	0.0217 (6)	−0.0032 (6)	0.0094 (6)	−0.0037 (5)
O5	0.0251 (7)	0.0254 (7)	0.0403 (8)	0.0007 (5)	−0.0099 (6)	0.0103 (6)
O6	0.0480 (11)	0.0316 (9)	0.1273 (19)	−0.0056 (8)	−0.0526 (12)	0.0061 (10)
O7	0.0661 (12)	0.0602 (11)	0.0298 (8)	−0.0355 (9)	−0.0036 (8)	−0.0023 (7)
O8	0.0378 (9)	0.0433 (9)	0.0561 (10)	−0.0220 (7)	0.0039 (7)	−0.0106 (8)
O9	0.0298 (7)	0.0194 (6)	0.0205 (6)	−0.0013 (5)	−0.0032 (5)	0.0045 (5)
C1	0.0158 (7)	0.0157 (7)	0.0171 (7)	0.0003 (6)	0.0003 (6)	0.0000 (6)
C2	0.0172 (8)	0.0178 (8)	0.0141 (7)	−0.0019 (6)	−0.0004 (6)	0.0013 (6)
C3	0.0149 (7)	0.0177 (8)	0.0154 (7)	−0.0014 (6)	0.0007 (6)	−0.0014 (6)
C4	0.0158 (7)	0.0157 (7)	0.0160 (7)	−0.0004 (6)	0.0010 (6)	0.0004 (6)
C5	0.0222 (8)	0.0180 (8)	0.0137 (7)	−0.0007 (6)	0.0016 (6)	0.0014 (6)
C6	0.0215 (8)	0.0164 (8)	0.0150 (7)	−0.0006 (6)	0.0001 (6)	−0.0030 (6)
C7	0.0215 (8)	0.0159 (7)	0.0140 (7)	−0.0020 (6)	−0.0006 (6)	0.0018 (6)
C8	0.0336 (10)	0.0275 (9)	0.0173 (8)	0.0032 (7)	−0.0001 (7)	−0.0071 (7)
C9	0.0172 (8)	0.0167 (7)	0.0169 (7)	−0.0003 (6)	0.0007 (6)	0.0001 (6)
C10	0.0433 (11)	0.0146 (8)	0.0260 (9)	−0.0018 (7)	0.0029 (8)	0.0046 (7)
C11	0.0185 (8)	0.0154 (7)	0.0168 (8)	−0.0009 (6)	0.0033 (6)	−0.0005 (6)
C12	0.0186 (8)	0.0145 (7)	0.0231 (8)	0.0004 (6)	0.0032 (6)	0.0013 (6)
C13	0.0186 (8)	0.0186 (8)	0.0257 (9)	−0.0006 (6)	0.0018 (7)	0.0018 (6)
C14	0.0203 (8)	0.0187 (8)	0.0270 (9)	−0.0035 (6)	0.0013 (7)	−0.0009 (7)
C15	0.0260 (9)	0.0142 (8)	0.0266 (9)	−0.0009 (6)	0.0028 (7)	−0.0003 (6)
C16	0.0208 (8)	0.0163 (8)	0.0197 (8)	0.0010 (6)	0.0021 (6)	0.0027 (6)
C17	0.0234 (9)	0.0232 (9)	0.0377 (11)	0.0000 (7)	−0.0063 (8)	0.0000 (8)
C18	0.0290 (10)	0.0345 (11)	0.0404 (11)	0.0064 (8)	−0.0097 (9)	0.0107 (9)
C19	0.0344 (11)	0.0194 (9)	0.0345 (10)	−0.0007 (7)	−0.0072 (8)	−0.0004 (8)
C20	0.076 (2)	0.084 (2)	0.0432 (15)	−0.0364 (17)	−0.0131 (14)	−0.0089 (14)
C21	0.0191 (8)	0.0162 (8)	0.0164 (7)	0.0001 (6)	0.0031 (6)	0.0006 (6)

### Geometric parameters (Å, °)

**Table:**

O1—C7	1.334 (2)	C8—H8A	0.9800
O1—C8	1.447 (2)	C8—H8B	0.9800
O2—C7	1.201 (2)	C8—H8C	0.9800
O3—C9	1.334 (2)	C10—H10A	0.9800
O3—C10	1.453 (2)	C10—H10B	0.9800
O4—C9	1.200 (2)	C10—H10C	0.9800
O5—C17	1.320 (2)	C11—C16	1.398 (2)
O5—C18	1.454 (2)	C11—C12	1.396 (2)
O6—C17	1.197 (2)	C11—C21	1.494 (2)
O7—C19	1.315 (3)	C12—C13	1.391 (2)
O7—C20	1.463 (3)	C12—H12	0.9500
O8—C19	1.186 (3)	C13—C14	1.399 (2)
O9—C21	1.220 (2)	C13—C17	1.495 (2)
C1—C2	1.393 (2)	C14—C15	1.394 (2)
C1—C6	1.395 (2)	C14—C19	1.508 (2)
C1—C21	1.500 (2)	C15—C16	1.388 (2)
C2—C3	1.387 (2)	C15—H15	0.9500
C2—H2	0.9500	C16—H16	0.9500
C3—C4	1.402 (2)	C18—H18A	0.9800
C3—C7	1.503 (2)	C18—H18B	0.9800
C4—C5	1.394 (2)	C18—H18C	0.9800
C4—C9	1.496 (2)	C20—H20A	0.9800
C5—C6	1.390 (2)	C20—H20B	0.9800
C5—H5	0.9500	C20—H20C	0.9800
C6—H6	0.9500		
			
C7—O1—C8	116.05 (14)	C16—C11—C12	119.28 (15)
C9—O3—C10	114.72 (13)	C16—C11—C21	118.36 (15)
C17—O5—C18	115.37 (15)	C12—C11—C21	122.33 (14)
C19—O7—C20	111.96 (19)	C13—C12—C11	120.56 (15)
C2—C1—C6	119.69 (15)	C13—C12—H12	119.7
C2—C1—C21	117.35 (14)	C11—C12—H12	119.7
C6—C1—C21	122.89 (14)	C12—C13—C14	119.71 (16)
C3—C2—C1	120.55 (14)	C12—C13—C17	121.00 (15)
C3—C2—H2	119.7	C14—C13—C17	119.28 (16)
C1—C2—H2	119.7	C15—C14—C13	119.97 (16)
C2—C3—C4	119.83 (14)	C15—C14—C19	118.98 (15)
C2—C3—C7	117.18 (14)	C13—C14—C19	121.01 (16)
C4—C3—C7	122.91 (14)	C16—C15—C14	120.04 (15)
C5—C4—C3	119.43 (14)	C16—C15—H15	120.0
C5—C4—C9	121.71 (14)	C14—C15—H15	120.0
C3—C4—C9	118.77 (14)	C15—C16—C11	120.44 (15)
C6—C5—C4	120.53 (14)	C15—C16—H16	119.8
C6—C5—H5	119.7	C11—C16—H16	119.8
C4—C5—H5	119.7	O6—C17—O5	123.51 (18)
C5—C6—C1	119.84 (14)	O6—C17—C13	123.94 (18)
C5—C6—H6	120.1	O5—C17—C13	112.54 (15)
C1—C6—H6	120.1	O5—C18—H18A	109.5
O2—C7—O1	125.28 (15)	O5—C18—H18B	109.5
O2—C7—C3	124.21 (15)	H18A—C18—H18B	109.5
O1—C7—C3	110.31 (13)	O5—C18—H18C	109.5
O1—C8—H8A	109.5	H18A—C18—H18C	109.5
O1—C8—H8B	109.5	H18B—C18—H18C	109.5
H8A—C8—H8B	109.5	O8—C19—O7	123.63 (19)
O1—C8—H8C	109.5	O8—C19—C14	124.31 (19)
H8A—C8—H8C	109.5	O7—C19—C14	112.02 (17)
H8B—C8—H8C	109.5	O7—C20—H20A	109.5
O4—C9—O3	124.16 (15)	O7—C20—H20B	109.5
O4—C9—C4	123.26 (15)	H20A—C20—H20B	109.5
O3—C9—C4	112.55 (13)	O7—C20—H20C	109.5
O3—C10—H10A	109.5	H20A—C20—H20C	109.5
O3—C10—H10B	109.5	H20B—C20—H20C	109.5
H10A—C10—H10B	109.5	O9—C21—C11	120.40 (14)
O3—C10—H10C	109.5	O9—C21—C1	119.80 (15)
H10A—C10—H10C	109.5	C11—C21—C1	119.80 (13)
H10B—C10—H10C	109.5		
			
C6—C1—C2—C3	3.2 (2)	C12—C13—C14—C15	−0.5 (3)
C21—C1—C2—C3	−179.84 (14)	C17—C13—C14—C15	−178.97 (17)
C1—C2—C3—C4	−0.8 (2)	C12—C13—C14—C19	177.06 (17)
C1—C2—C3—C7	−177.49 (15)	C17—C13—C14—C19	−1.4 (3)
C2—C3—C4—C5	−2.4 (2)	C13—C14—C15—C16	−0.4 (3)
C7—C3—C4—C5	174.02 (15)	C19—C14—C15—C16	−177.95 (17)
C2—C3—C4—C9	174.17 (14)	C14—C15—C16—C11	0.5 (3)
C7—C3—C4—C9	−9.4 (2)	C12—C11—C16—C15	0.2 (2)
C3—C4—C5—C6	3.4 (2)	C21—C11—C16—C15	178.04 (15)
C9—C4—C5—C6	−173.13 (15)	C18—O5—C17—O6	−1.2 (3)
C4—C5—C6—C1	−1.0 (2)	C18—O5—C17—C13	179.94 (17)
C2—C1—C6—C5	−2.3 (2)	C12—C13—C17—O6	−162.3 (2)
C21—C1—C6—C5	−179.05 (15)	C14—C13—C17—O6	16.2 (3)
C8—O1—C7—O2	0.9 (2)	C12—C13—C17—O5	16.6 (3)
C8—O1—C7—C3	175.88 (13)	C14—C13—C17—O5	−164.95 (17)
C2—C3—C7—O2	103.63 (19)	C20—O7—C19—O8	14.0 (3)
C4—C3—C7—O2	−72.9 (2)	C20—O7—C19—C14	−168.1 (2)
C2—C3—C7—O1	−71.44 (18)	C15—C14—C19—O8	73.0 (3)
C4—C3—C7—O1	112.03 (17)	C13—C14—C19—O8	−104.6 (2)
C10—O3—C9—O4	−0.5 (2)	C15—C14—C19—O7	−104.9 (2)
C10—O3—C9—C4	177.76 (14)	C13—C14—C19—O7	77.6 (2)
C5—C4—C9—O4	162.70 (17)	C16—C11—C21—O9	−25.3 (2)
C3—C4—C9—O4	−13.8 (2)	C12—C11—C21—O9	152.50 (16)
C5—C4—C9—O3	−15.5 (2)	C16—C11—C21—C1	155.19 (15)
C3—C4—C9—O3	167.94 (14)	C12—C11—C21—C1	−27.0 (2)
C16—C11—C12—C13	−1.0 (2)	C2—C1—C21—O9	−29.0 (2)
C21—C11—C12—C13	−178.79 (15)	C6—C1—C21—O9	147.90 (17)
C11—C12—C13—C14	1.2 (3)	C2—C1—C21—C11	150.56 (15)
C11—C12—C13—C17	179.64 (16)	C6—C1—C21—C11	−32.6 (2)
